# The impact of atopy on the clinical characteristics of mycoplasma pneumoniae pneumonia in pediatric patients

**DOI:** 10.1038/s41598-024-84557-z

**Published:** 2025-01-20

**Authors:** Yujie Qin, Yuxia Yang, Junxiang Li, Jun Guan

**Affiliations:** Third Affiliated Hospital of Zhengzhou University, Zhengzhou, China

**Keywords:** Mycoplasma pneumoniae pneumonia, Atopy, Children, Severe pneumonia, Bronchiolitis, Microbiology, Diseases, Risk factors

## Abstract

Mycoplasma pneumoniae (MP) is one of the pathogens that cause community-acquired pneumonia in children. Atopic diseases are also common in children. However, the impact of atopy on Mycoplasma pneumoniae pneumonia (MPP) in children is still unclear. The purpose of this study is to analyze the impact of atopy on the clinical characteristics of MPP in children, and provide a diagnosis and treatment plan. A total of 489 children hospitalized for MPP in our hospital from June 2023 to December 2023 were selected. They were divided into an atopic group (*n* = 172) and a non-atopic group (*n* = 317) based on whether they had atopy or not. Clinical data, treatment regimens, and laboratory indicators were compared between the two groups. Eosinophil count, lactate dehydrogenase and IgE levels were higher in the atopic group than in the non-atopic group. Additionally, neutrophil percentage, procalcitonin levels were lower in the atopic group than in the non-atopic group (*P* < 0.05). The proportion of bronchiolitis type on lung imaging was higher in the atopic group, and there was a higher incidence of severe pneumonia compared to the non-atopic group (*P* < 0.05). Atopy may lead to severe MPP and bronchiolitis-type MPP. Therefore, the treatment and prognosis of these children should be given more attention.

## Introduction

Mycoplasma pneumoniae (MP) is a special pathogen that not only can penetrate host cell membranes and invade the respiratory tract directly to cause lung damage, but also can cause damage to other organs through abnormal immune responses. In recent years, the incidence of Mycoplasma pneumoniae pneumoniae (MPP) has gradually increased, accounting for 20–40% of community-acquired pneumonia in children over 5 years old, and occurring every 3–7 years in regional outbreaks^1^. Atopy refers to a hereditary tendency for the body to produce immunoglobulin E (IgE) type immune responses to common environmental allergens^2^. Some studies have shown that MP infection is one of the risk factors that cause allergic diseases and induce allergy^3^. After children are infected with MP, their immune regulation is disrupted, and the interaction with atopy increases the risk of severe Mycoplasma pneumoniae pneumonia (SMPP)^4^. Therefore, this study aims to provide reference for the diagnosis and treatment of atopic children with MPP by analyzing the differences in clinical data and laboratory indicators between atopic and non-atopic MPP patients.

## Materials and methods

### Study objects

The study included 595 children with MPP who were hospitalized at the Third Affiliated Hospital of Zhengzhou University from June 2023 to December 2023.According to the exclusion criteria, 106 cases were excluded, and 489 children with MPP were finally included as the research objects. Inclusion criteria: (1) the younger than 14 years old; (2) MPP was diagnosed according to the “Guidelines for the Diagnosis and Treatment of Mycoplasma Pneumoniae Pneumonia in Children (2023 Edition)^5^”: having respiratory symptoms such as fever, cough, wheezing, and difficulty breathing; pulmonary imaging demonstrated pneumonia; combined with at least one of the following criteria, single serum antibody titer ≥ 1:160 or double serum antibody titer increased more than 4 times during the course of disease; Nasopharyngeal swab MP-DNA or MP-RNA positive; Exclusion criteria: (1) patients with respiratory diseases such as bronchial foreign body, tuberculosis, bronchopulmonary dysplasia, and respiratory tract malformation; (2) patients with autoimmune diseases, tumors or immunodeficiency diseases; (3) those with serious heart, liver, kidney disease; (4) those who have long-term use of corticosteroids or immunosuppressants; (5) patients admitted to hospital in the recovery stage of MPP; (6) patients with incomplete clinical data. This study was approved by the Ethics Committee of the Third Affiliated Hospital of Zhengzhou University (Ethics Approval No: 2024-113-01). Since this study only collects clinical data from children without their names and other identifying information, it does not affect the health and rights of the children. Therefore, we applied for informed consent from the Ethics Committee of the Third Affiliated Hospital of Zhengzhou University and obtained approval. This study follows the Declaration of Helsinki. All methods used in this study were conducted in accordance with relevant guidelines and regulations.

### Grouping

Based on the presence or absence of atopy, the children were categorized into an atopic group of 172 cases and a non-atopic group of 317 cases. We define atopic as the presence of any one of the following conditions: (1) presence of clinically diagnosed allergic diseases, such as atopic rhinitis, atopic dermatitis, atopic asthma, etc. (2) with a confirmed food or drug allergy history; (3) positive results in food stimulation test; (4) at least two or more indicators of atopy: ① positive skin prick test; ② at least one kind of positive serum allergen: specific IgE ≧ 0.35kU/L; ③increased total IgE.

### Study methods

The demographic characteristics (gender, age), clinical manifestations (the length of hospital stay, the duration of fever, the presence of wheezing, the duration of pulmonary rales, oxygen therapy), laboratory indicators, pulmonary imaging findings and treatment regimens of the children in the atopic group and non-atopic group were compared.

### Statistical analysis

Perform statistical analysis on the data using SPSS 26.0. Normally distributed data were expressed as means ± standard deviation($\:\stackrel{-}{x}$ ± *s*), and the comparison between the two groups was performed by independent sample *t* test; Non-normally distributed data were expressed as the median (interquartile ranges: P_25_, P_75_), and the Mann Whitney U test was used for the comparison between the two group. The count data is represented by composition ratio (%), and the comparison between two groups is conducted using χ^2^ test, or Fisher exact probability method. *P* < 0.05 was considered statistically significant.

## Results

### Comparison of demographic characteristics

The average age of the atopic group was (6.48 ± 2.55) years old, including 90 boys and 82 girls. The non-atopic group was (6.4 ± 2.65) years old, with 172 boys and 145 girls. According to age, the enrolled children were divided into < 1 years old group, 1– < 3 years old group, 3– < 6 years old group, and ≥ 6 years old group. The children in both groups were mainly ≥ 6 years old, and there were no statistical differences in the distribution of gender and age between the two groups (*P* > 0.05) (Table 1).

**Table 1:** Comparison of demographic characteristics and clinical manifestations of patients with MPP.

Characteristics	Atopic group (*n* = 172)	Non-atopic group (*n* = 317)	Z/χ^2^	*P*
Demographic characteristics				
Gender, n (%)				
Boys	90 (52.3%)	172 (54.3%)	0.168	0.682
Girls	82 (47.7%)	145 (45.7%)		
Age, n (%)				
< 1 years	1 (0.6%)	10 (3.2%)	4.150	0.246
1–< 3 years	16 (9.3%)	27 (8.5%)		
3–< 6 years	51 (29.7%)	81 (25.6%)		
≥ 6 years	104 (60.5%)	199 (62.8%)		
Demographic characteristics				
Wheezing, n(%)				
Yes	43 (25%)	40 (12.6%)	12.13	< 0.001
No	129 (75%)	277 (87.4%)		
Oxygen therapy, n(%)				
Yes	9 (5.2%)	9 (2.8%)	1.802	0.180
No	163 (94.8%)	308 (97.2%)		
The length of hospital stays, d	6 (5, 10)	6 (5.5, 10)	0.517	0.605
The duration of fever, d	0.5 (0, 2)	1 (0, 2)	0.512	0.609
The duration of pulmonary rales, d	3 (0.25, 5)	2 (0, 5)	2.200	0.026

### Comparison of clinical manifestations

The atopic group was more prone to wheezing than the non-atopic group, and the duration of pulmonary rales was longer (*P* < 0.05). However, there was no statistical difference between the two groups in the length of hospital stay, the duration of fever and incidence of oxygen therapy (*P* > 0.05) (Table 1).

### Comparison of laboratory indicator

There were no significant differences in the overall distribution of white blood cell count, neutrophil and lymphocyte counts, between the atopic group and the non-atopic group, but there were statistically significant differences in neutrophil percentage (N%), neutrophil to lymphocyte percentage (N%/L%), eosinophil count (EO) and NK cell levels between the atopic group and the non-atopic group (*P* < 0.05). At the same time, there were no differences in the distribution of infection indicators such as C-reactive protein(CRP), erythrocyte sedimentation rate (ESR), ferritin and D-dimer between the two groups, but the lactate dehydrogenase (LDH) level in the atopic group was higher than that in the non-atopic group, and the procalcitonin (PCT) was lower than that in the non-atopic group, and there were statistical differences between the two groups (*P* < 0.05). In addition, the total IgE level in the atopic group was higher than that in the non-atopic group, and the difference between the two groups was statistically significant (*P* < 0.05) (Table 2).

**Table 2:** Comparison of Laboratory Indicator of patients with MPP.

Characteristics	Atopic group (*n* = 172)	Non-atopic group (*n* = 317)	Ζ	*P*
WBC (×10^9^/L)	7.56 (6.34, 9.86)	7.76 (6.03, 9.65)	2.640	0.792
N%	63.8 (54.7, 70.6)	66.3 (54.9, 72.8)	2.494	0.013
L%	26.4 (21.0, 35.3)	24.4 (18.8, 36.1)	1.745	0.081
N%/L%	2.40 (1.57, 3.33)	2.69 (1.49, 3.88)	1.999	0.046
N (×10^9^/L)	5.00 (3.92, 6.61)	5.03 (3.48, 6.76)	0.589	0.556
L (×10^9^/L)	2.15 (1.58, 3.11)	1.93 (1.39, 2.86)	1.188	0.235
EO%	1.3 (0.4, 3.3)	0.5 (0.1, 1.4)	4.709	< 0.001
EO (×10^9^/L)	0.12 (0.04, 0.30)	0.04 (0.01, 0.11)	4.661	< 0.001
CRP (mg/L)	10.08 (4.19, 17.8)	9.98 (4.75, 18.57)	0.707	0.480
PCT (ng/L)	0.042 (0, 0.069)	0.050 (0, 0.113)	2.712	0.007
ESR (mm/h)	28 (20, 39)	29 (21, 39)	0.250	0.802
Ferritin (ug/L)	99.9 (74.5, 122.0)	105.0 (77.7, 154.5)	1.882	0.060
D-dimer (mg/L)	0.20 (0.14, 0.28)	0.21 (0.15, 0.30)	1.484	0.138
LDH (U/L)	243.6 (219.6, 279.2)	239.4 (214.8, 268.9)	1.966	0.049
ALT (U/L)	13.3 (10.6, 16.5)	13.2 (10.9, 17.0)	0.412	0.681
AST (U/L)	27.8 (23.4, 32.5)	27.4 (23.0, 34.0)	0.449	0.653
Urea (mmol/L)	3.35 (2.80, 3.99)	3.29 (2.73, 3.94)	0.560	0.575
Creatinine (µmol/L)	32.2 (27.4, 37.5)	32.2 (27.5, 37.0)	0.144	0.886
CD3,%	67.8 (62.5, 72.1)	67.3 (61.9, 71.8)	1.134	0.257
CD4,%	36.3 (31.7, 40.4)	36.1 (30.7, 41.5)	0.087	0.931
CD8,%	2.1 (20.9, 29.2)	24.8 (21.1, 28.2)	0.628	0.530
CD4/CD8	1.4 (1.2, 1.9)	1.5 (1.2, 1.8)	0.461	0.645
B%	17.8 (14.2, 22.3)	17.3 (13.5, 21.9)	0.817	0.414
NK,%	11.1 (7.4, 15.5)	12.5 (8.5, 16.8)	2.525	0.024
IgG (g/L)	9.78 (8.25, 11.23)	9.56 (7.97, 11.2)	0.559	0.576
IgA (g/L)	1.27 (0.95, 1.67)	1.27 (0.80, 1.64)	1.008	0.313
IgM (g/L)	1.22 (0.96, 1.55)	1.18 (0.89, 1.55)	1.296	0.195
IgE (IU/mL)	79.0 (27.2, 302.6)	31.0 (18.8, 101.0)	5.193	< 0.001

Note: WBC represents white blood cell count; N% is the percentage of neutrophils; L% is the percentage of lymphocytes; N%/L% is neutrophil to lymphocyte percentage; N is the count of neutrophils; L is the count of lymphocytes; EO% is the percentage of eosinophils; EO is the count of eosinophils; CRP is a C-reactive protein; ESR is erythrocyte sedimentation rate; PCT is procalcitonin; LDH is a lactate dehydrogenase; IgG is immunoglobulin G; IgA is immunoglobulin A; IgM is immunoglobulin M; IgE is immunoglobulin E.

### Pulmonary imaging examination

According to chest CT images, they were divided into 4 categories^6^, including patchy infiltration type, bronchiolitis type (unilateral or bilateral tree bud sign, central lobular nodule, thickening of bronchial wall), pulmonary consolidation type, and mixed type. The bronchiolitis type was dominated by atopic group, while the lung solid type and spot exudation type were dominated by non-atopic group. There were statistical differences in the distribution of atopic group and non-atopic group in the imaging findings of the four groups (*P* < 0.001) (Table 3).

**Table 3:** Pulmonary imaging examination of patients with MPP.

	Patchy infiltration type	Bronchiolitis type	Pulmonary consolidation type	Mixed type	χ^2^	*P*
Atopic group (*n* = 172)	17 (9.9%)_a_	65 (37.8%)_b_	52 (30.2%)_a_	38 (22.1%)_b_	47.242	< 0.001
non-atopic group (*n* = 317)	65 (20.5%)_a_	52 (16.4%)_b_	162 (51.1%)_a_	38 (12%)_b_		

Note: Different subscripts indicate statistical differences between the two groups(*P* < 0.05).

### Severity of pneumonia

**Fig. 1:**
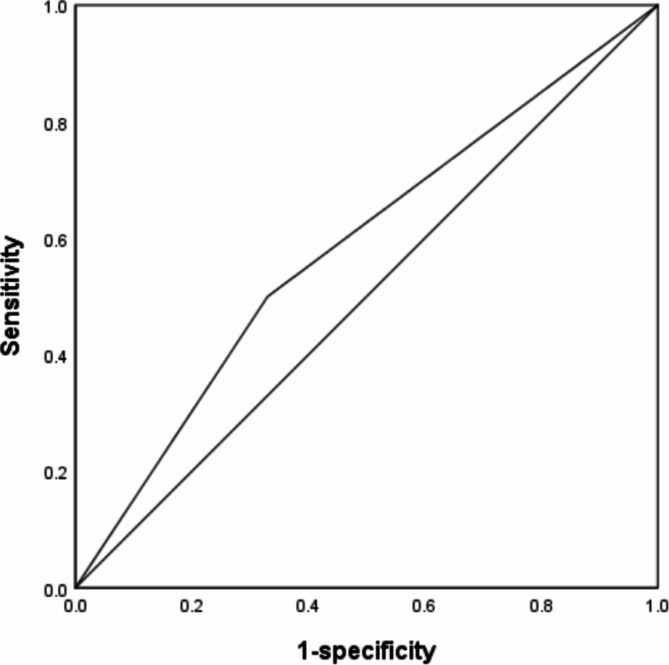
Prediction curve of atopic dermatitis for SMPP.

According to the “Guidelines for the Diagnosis and Treatment of Mycoplasma Pneumoniae Pneumonia in Children (2023 Edition)^5^” criteria for severe cases, the included children were divided into mild and severe groups. The results of statistical analysis showed that severe pneumonia accounted for a larger proportion of children in atopic group, and there was a statistical difference between the two groups (*P* < 0.05) (Table 4). Logistic regression was conducted with atopic as the independent variable and pneumonia severity as the dependent variable. The results showed that atopy was a risk factor for the occurrence of SMPP (Table 5). The ROC curve showed a sensitivity of 50%, specificity of 67%, and AUC = 0.585 (*95% CI* 0.507–0.662) for predicting SMPP (Fig. 1).

**Table 4:** Severity of pneumonia and treatment of patients with MPP.

Characteristics	Atopic group (*n* = 172)	Non-atopic group (*n* = 317)	χ^2^/Ζ	*P*
Severity of pneumonia				
Mild groups	141 (82%)	286 (90.2%)	6.845	0.009
Severe groups	31 (18%)	31 (9.8%)		
Treatment				
Duration of methylprednisolone use, d	4 (2,6)	4 (2,5)	3.393	0.001
Intravenous methylprednisolone, n(%)	130 (75.6%)	224 (77%)	0.120	0.729
Bronchoscopy, n(%)	35 (20.3%)	51 (16.1%)	1.396	0.237
Human immunoglobulin, n(%)	1 (0.6%)	3 (0.9%)	0.183	0.669

**Table 5:** Univariate logistic regression of SMPP.

	b	Wald χ^2^	*P*	OR	95%CI
Atopy	0.707	6.660	0.010	2.028	1.185–3.471

### Treatment

All children with MPP were treated with macrolide antibiotics. Some macrolide resistant children were replaced with tetracycline antibiotics. Patients with severe inflammatory reactions should be treated with intravenous corticosteroids, namely methylprednisolone; Patients with large lung consolidation area and atelectasis should undergo bronchoscopy and bronchoalveolar lavage treatment; There were many complications, and those with severe conditions were treated with human immunoglobulin. There was no statistically significant difference (*P* > 0.05) in the proportion of methylprednisolone, bronchoscopy, and human immunoglobulin use between the atopic group and the non-atopic group, but the atopic group had a longer duration of methylprednisolone use (Table 4).

## Discussion

This study demonstrated that there were no differences in age and gender distribution between children with atopic and non-atopic conditions. The onset of MPP primarily occurred in school-age children, with a noticeable trend also observed in infants. There were no statistically significant disparities in hospitalization time and the duration of fever between the two groups of children, but the atopic group exhibited a higher incidence of wheezing, longer duration of pulmonary rales, as well as elevated levels of eosinophils and IgE compared to the non-atopic group. Shenghua et al.^7^ indicated that recurrent wheezing in children with MP infection was closely related to allergens, allergy history, and atopic constitution. Fan et al.^8^ also affirmed that atopy plays an important role in children’s wheezing and coughing. Some studies suggest^9^ that MP antigen stimulates the body to produce autoantibodies, while the body activates the complement system to release inflammatory factors, causing an imbalance in the THL/TH2 ratio and stimulating B cells to produce IgE and increase the synthesis and secretion of eosinophils, resulting in wheezing^10^. Excessive inflammatory response due to atopy among children with MP infection leads to sustained tissue inflammation and damage, causing severe immunopathological processes and pathological damage, resulting in recurrent wheezing, prolonged coughing and wheezing, and prolonged rales, ultimately leading to prolonged hospitalization.

Lymphocytes and neutrophils are an important part of the body’s immune response.MP infection triggers the activation of neutrophils to phagocytose pathogen, while leading to the activation and release of lymphocytes^11^. Lymphocytes are divided into T lymphocytes and B lymphocytes, which participate in cellular and humoral immune responses, respectively. Atopic children have immune disorders, leading to increased lymphocyte expression. The results demonstrated that the lymphocyte count, CD3, CD4, CD8, and B lymphocyte levels in the atopic group were higher than those in the non-atopic group, but there was no statistical difference between the two groups. NLR and neutrophil counts are recognized as parameters for disease progression in MPP^12,13^. The findings of this study indicated that there were no statistically significant differences in NLR and neutrophil count between the two groups; however, there were statistical differences in the neutrophil percentage and its ratio to the lymphocyte percentage between the two groups, which might be associated with the absence of neutrophil and lymphocyte counts in some children. It is also worth noting that research results have shown a close relationship between NK cells and the occurrence and progression of MPP^14^. This study found that the level of NK cells in the atopic group is lower than that in the non-atopic group. Nevertheless, further exploration is required as the underlying mechanism remains unknown.

PCT is an immunomodulatory protein found in the human body, primarily transcribed and generated in parafollicular cells of the thyroid gland and secreted in small amounts by tissue cells such as the liver, kidneys, and lungs. It exhibits high sensitivity and specificity to bacterial infections but does not elevate during viral infections or allergic reactions caused by various factors. This phenomenon may be associated with the release of certain interferons that reduce the up-regulation of IL1β on PCT. Studies have confirmed that although procalcitonin only shows a slight increase after MP infection, it still holds diagnostic value^15,16^. In this study, children infected with MP showed a slight increase in procalcitonin levels, while the atopic group exhibited lower levels compared to the non-atopic group.

LDH is widely present in various tissues of the body such as the brain, kidneys, liver, myocardium, and lungs. When cells are invaded by pathogens, the cell membrane is damaged, and large amounts of LDH are released into the extracellular space, causing a marked increase in serum LDH^17^. Therefore, LDH, as a marker of tissue damage, is a warning indicator for SMPP^18–21^. The results of this study showed that the lactate dehydrogenase levels of children with atopic group were higher than those of children with non-atopic group. Zhihua et al.^22^ also confirmed this. Compared to children with non-atopic constitution, children with atopic constitution have more severe disease, greater inflammatory response, and therefore, their serum LDH level increases more markedly, which can also serve as a basis for systemic administration of glucocorticoids^23^. There was no difference in the use rate of glucocorticoids between the two groups in the study, but the duration of glucocorticoid application was longer in the atopic group. Glucocorticoids have anti-inflammatory, anti-atopic, and immune-suppressive effects and are the most effective anti-inflammatory drugs currently available. Children with atopic constitution have high airway reactivity and prolonged wheezing, which should be taken seriously and given targeted treatment.

This study found that the pulmonary imaging of children in the atopic group mainly showed bronchiolitis type, while children in the non-atopic group mainly showed consolidation type. Xufeng et al.^24^ found that the imaging changes of MPP showing bronchiolitis type are closely related to atopic constitution. Kunling et al.^25^ also stated that MP bronchiolitis often occurs in infants and preschool children with a family or personal history of allergies. This may be related to the excessive immune response in children with atopic constitution. MP adheres to respiratory epithelial cells through P1 adhesin, activates the body’s immune response, induces the production of pro-inflammatory factors by its own epithelial cells, and secretes Community Acquired Respiratory Distress Syndrome (CARDS) toxin^21,26^. Krishnan et al.^27^ found that CARDS toxins can cause non programmed cell death in targeted cells, while the host initiates an innate immune response, damaging the structure of bronchioles. This abnormal repair process leads to fibrous tissue proliferation, causing luminal stenosis, and in severe cases, even leading to the occurrence of obstructive bronchiolitis. In recent years, with the increase of MP infection rate, there has been an increasing trend of MP causing bronchiolitis, and the probability of residual obstructive bronchiolitis is also gradually increasing, which should be fully valued by clinical physicians and early application of glucocorticoids should be given.

## Conclusion

In summary, children with atopic constitution often have wheezing caused by MPP, which lasts for a long time. The imaging findings of bronchiolitis type and the incidence of severe MPP are higher in the atopic group. When there is a high suspicion of MP infection in a child, timely screening and personalized treatment should be given. If necessary, early and sufficient treatment with glucocorticoids should be administered to avoid the occurrence of SMPP. The data collection window for this study is relatively narrow, and further validation with large sample and multi center data is needed.

## Data Availability

All data can be obtained by contacting the corresponding authors.

## References

[1] Xin, W. & Deyu, Z. Research progress on molecular epidemiology of Mycoplasma pneumoniae in children. *Int. J. Pediatr.* **2021**, 488–491. 10.3760/cma.j.issn.1673-4408.2021.07.012 (2021).

[2] Natalie, M. D. C., Mario, A. S. B., Dennis, K. L. & Atopy A collection of comorbid conditions. *J. Allergy Clin. Immunol. Pract.* **2021**, 9. 10.1016/j.jaip.2021.09.002 (2021). 10.1016/j.jaip.2021.09.002 34509674

[3] Qing, Y., Jian-Hua, M., Qiang, S. & Shi-Qiang, S. Mycoplasma pneumoniae induces allergy by producing P1-specific immunoglobulin E. *Ann. Allergy Asthma Immunol.* **2018**, 121. 10.1016/j.anai.2018.03.014 (2018). 10.1016/j.anai.2018.03.014 29555351

[4] Wei, X. Child pneumonia mycoplasma pneumonia and respiratory failure. *Chin. J. Pract. Pediatr. Clin.* **33**, 887–891. 10.3760/cma.j.issn.2095-428X.2018.12.003 (2018).

[5] The National Health Commission of the People’s Republic of China. Diagnosis and treatment guidelines for mycoplasma pneumonia in children (2023 Edition). *Int. J. Epidemiol. Infect. Dis.* **50**, 79–85. 10.3760/cma.j.cn331340-20230217-00023 (2023).

[6] Cho, Y. J. et al. Correlation between chest radiographic findings and clinical features in hospitalized children with Mycoplasma pneumoniae pneumonia. *PloS One* **14**, e0219463 (2019). 31461462 10.1371/journal.pone.0219463 PMC6713385

[7] Shenghua, Q., Xiaohua, W. & Li, Z. Exploration of the relationship between wheezing in infants and young children and Mycoplasma pneumoniae infection. *Exp. Therapeut Med.* **18**, 1090–1093. 10.7499/j.issn.1008-8830.2016.11.007 (2016).

[8] Fan, Q. et al. Roles of T-cell immunoglobulin and mucin domain genes and toll-like receptors in wheezy children with Mycoplasma pneumoniae pneumonia. *Heart Lung Circ.* **25**, 1226–1231 (2016). 27185658 10.1016/j.hlc.2016.03.019

[9] Tartari, A. P. S. et al. Anti-inflammatory effect of ozone therapy in an experimental model of rheumatoid arthritis. *Inflammation* **43**, 985–993 (2020). 32382842 10.1007/s10753-020-01184-2

[10] Xiaoyan, Z., Tao & Pingping, W. T lymphocyte subsets, IL-9, and IFN in children with Mycoplasma pneumoniae pneumonia- γ the relationship between level and wheezing. *J. North. Sichuan Med. Coll.* **37**, 1463–1466. 10.3969/j.issn.1005-3697.2022.11.021 (2022).

[11] Haizhen, Y., Weiyuan, W. & Xiaoyu, L. Value of leukocyte count and lymphocyte subsets in diagnosis of mycoplasma pneumoniae infection in children. *J. Mol. Diagn. Therapy* **14**, 2040–2043. 10.3969/j.issn.1674-6929.2022.12.006 (2022).

[12] Xue, Z., Ruiyang, S., Wanyu, J., Peng, L. & Chunlan, S. A new dynamic nomogram for predicting the risk of severe Mycoplasma pneumoniae pneumonia in children. *Sci. Rep.* **14**, 2369. 10.1038/s41598-024-58784-3 (2024). 10.1038/s41598-024-58784-3 PMC11002011 38589453

[13] Dan, L. et al. Neutrophil-to-lymphocyte ratio as a predictor of poor outcomes of Mycoplasma pneumoniae pneumonia. *Front. Immunol.* **14**, 859. 10.3389/fimmu.2023.1302702 (2024). 10.3389/fimmu.2023.1302702 PMC10758472 38169689

[14] Pánisová, E., Unger, W. W., Berger, C. & Meyer Sauteur, P. M. Mycoplasma pneumoniae–specific IFN-γ–producing CD4 + effector-memory T cells correlate with pulmonary disease. *Am. J. Respir Cell. Mol. Biol.* **64**, 143–146 (2021). 33385212 10.1165/rcmb.2020-0237LE PMC7780996

[15] Yingjie, W., Xuemei, B., Zhengjuan, L., Yongli, Z. & Li, Z. Changes and significance of immune function, procalcitonin and C-reactive protein in children with mycoplasma pneumoniae pneumonia. *Chin. Pediatric Emerg. Med.* **2014**, 501–503 (2014).

[16] Haiyan, Q. Clinical significance of serum procalcitonin detection in children with mycoplasma pneumoniae pneumonia. *Chin. J. Pract. Med* **2019**, 39–41 (2019).

[17] Huaiping, Y., Kai, L., Wenya, L. & Xiaoyan, Z. Chest CT thin layer rebuild joint lactate dehydrogenase and c-reactive protein in the diagnosis of refractory mycoplasma pneumoniae pneumonia in children. *Chin. J. Med. Imaging* **31**, 375–378. 10.3969/j.issn.1005-5185.2023.04.015 (2023).

[18] Nan, M., Yazhou, J., Lie, Z. & Chunfeng, H. The clinical significance of lung ultrasound score combined with blood CRP and LDH levels in evaluating the degree of disease in children with mycoplasma pneumoniae pneumonia. *Chin. J. Ultrasound Med.* **37**, 993–997. 10.3969/j.issn.1002-0101.2021.09.011 (2021).

[19] Chunxia, C. Analysis of laboratory indexes and clinical characteristics of children with refractory mycoplasma pneumoniae pneumonia. *Maternal Child. Health Care China*. **34**, 1085–1088. 10.7620/zgfybj.j.issn.1001-4411.2019.05.39 (2019).

[20] Li, P., Wang, W., Zhang, X., Pan, J. & Gong, L. Observational retrospective clinical study on clinical features of macrolide-resistant Mycoplasma pneumoniae pneumonia in Chinese pediatric cases. *Sci. Rep.* **14**, 5632. 10.1038/s41598-024-55311-2 (2024). 38453960 10.1038/s41598-024-55311-2 PMC10920782

[21] Lin, T., Shumin, H., Chen, Z., Yuanyuan, Z. & Zhimin, C. Refractory mycoplasma pneumoniae pneumonia in children: early recognition and management. *J. Clin. Med.* **11**, 859. 10.3390/jcm11102824 (2022). 35628949 10.3390/jcm11102824 PMC9144103

[22] Zhihua, W., Yushui, W., Yan, L., Xin, W. & Yajuan, Z. Clinical characteristics and serum interleukin-17 levels of children with atopic constitutional mycoplasma pneumoniae pneumonia. *Chin. Clin. J. Pract. Pediatr.* **32**, 668–671. 10.3760/cma.j.issn.2095-428X.2017.09.008 (2017).

[23] Yan, Q. et al. Risk factors for delayed radiographic resolution in children with refractory Mycoplasma pneumoniae pneumonia. *J. Int. Med. Res.* **49**, 03000605211015579 (2021). 34034536 10.1177/03000605211015579 PMC8161875

[24] Xufeng, L., Xingjun, L., Jinming, G. & Haoxun, T. Clinical and bronchoscopic analysis of mycoplasma pneumoniae pneumonia with different imaging manifestations. *Beijing Med. Sci.* **44**, 857–860. 10.15932/j.0253-9713.2022.09.018 (2022).

[25] Kunling, H. et al. Clinical characteristics of mycoplasma pneumoniae bronchiolitis in children. *Chin. Clin. J. Pract. Pediatr.* **37**, 909–913. 10.3760/cma.j.cn101070-20210722-00861 (2022).

[26] XiaoLin, S. & Zhimin, C. Research progress of Mycoplasma pneumoniae pneumonia complicated with bronchiolitis obliterans. *Sci. Educ. Guide* **2019**, 41–42. 10.16400/j.cnki.kjdkz.2019.04.020 (2019).

[27] Krishnan, M., Kannan, T. & Baseman, J. B. Mycoplasma pneumoniae CARDS toxin is internalized via clathrin-mediated endocytosis. *PloS One* **8**, e62706 (2013). 23667510 10.1371/journal.pone.0062706 PMC3647021

